# Using Digital Platforms to Promote Blood Donation: Motivational and Preliminary Evidence from Latin America and Spain

**DOI:** 10.3390/ijerph18084270

**Published:** 2021-04-17

**Authors:** Joan Torrent-Sellens, Cristian Salazar-Concha, Pilar Ficapal-Cusí, Francesc Saigí-Rubió

**Affiliations:** 1 Faculty of Economics and Business, Universitat Oberta de Catalunya (UOC), 08035 Barcelona, Spain; jtorrent@uoc.edu (J.T.-S.); pficapal@uoc.edu (P.F.-C.); 2 Interdisciplinary Research Group on ICTs (i2TIC), Universitat Oberta de Catalunya (UOC), 08035 Barcelona, Spain; 3 Faculty of Economic and Administrative Sciences, Universidad Austral de Chile (UACh), Valdivia 5110566, Chile; cristiansalazar@uach.cl; 4 Faculty of Health Sciences, Universitat Oberta de Catalunya (UOC), 08018 Barcelona, Spain

**Keywords:** blood donation, digital platforms, collaborative exchanges, consumer behaviour, Theory of Planned Behaviour

## Abstract

The lack of blood donors is a global problem that prevents the demand for blood prompted by an ageing population and increased life expectancy from being met. The aim of this study was to conduct an initial exploration of the reasons for using digital platforms in blood donation. Using a Theory of Planned Behaviour (TPB) framework, microdata for 389 participants from Latin American countries and Spain, and Partial Least Square-Structural Equation Modelling (PLS-SEM), the study obtained three main prediction paths. The first two started from feelings of trust in the digital community and a positive mood state associated with a modern lifestyle, and they were linked to attitudes and behavioural control in the explanation of the intention to donate and actual blood donation. The third path started from modern lifestyles, and was linked to the subjective norm in the prediction of intention and actual donation. These paths represent one of the very first attempts to predict intentions of donation and collaborative donation by taking a PLS-SEM approach. By determining the paths underpinning collaborative blood donors’ motives, the results of this study provide strong support for the usefulness of the TPB model within the context of digital platform use and blood donation.

## 1. Introduction

The ageing population and the rise in life expectancy are together increasing the demand for blood supplies. Having enough blood and blood components available to meet the demand depends on the blood donation rate and frequency [1,2]. The lack of blood donors is a global problem [3,4]. In Japan, for example, the number of blood donations is expected to fall from 5,260,000 to 4,770,000 (9.3%) between 2012 and 2025 [5]. This drop is associated mainly with donors in the 20 to 30-year-old age group. Furthermore, it is estimated that, by 2025, 5,660,000 donations will be required. This represents a 15.7% shortfall in donations for that year, and the percentage is expected to more than double by 2050 [5]. Germany and the United States are also expected to experience similar difficulties recruiting and retaining an adequate number of blood donors, which is only made worse by the ageing population and by ever stricter donor selection criteria [6]. 

This scarcity becomes alarming in the face of natural disasters and tragedies, or of epidemics and pandemics. During the Severe Acute Respiratory Syndrome (SARS) outbreak in mid-2003, in China, a drop in the volume of blood collected was detected [7]. Today, the social restrictions placed on everyone to slow down the spread of SARS-CoV-2 are an added challenge to ensuring the availability of sufficient blood supplies [8,9,10,11]. Within this context of health crisis and mobility restrictions, the need to recruit and retain new donors has been highlighted [12]. The World Health Organization (WHO) specifies a minimum of 10 donations per 1000 inhabitants [13]. The rate per 1000 inhabitants currently stands at 31.5 donations in high income countries, 15.9 in upper-middle income countries, 6.8 in lower-middle income countries, and 5 in low income countries. Thus, of the 107 million donations collected worldwide, the average annual number of donations per blood centre is 3100 in low and middle income countries, compared to 15,000 in high income countries [13]. 

Every year, blood banks employ different strategies to recruit and retain blood donors (promotional activities, posters and leaflets) [14]. However, such recruitment has had limited success insofar as voluntary, unpaid blood donations are concerned. In 79 countries, more than 90% of blood supplies come from voluntary donors, whereas in 56 countries, more than 50% of supplies come from relatives or paid donors [13]. Thus, an understanding of the factors that motivate or dissuade people from donating blood is crucial to the development of blood donation and collection strategies [15]. 

Various strategies have considered using information and communication technologies in general, and Internet-based smart phones in particular, to promote, motivate, recruit and retain blood donors. Simple approaches such as sending text messages (SMS, short message service), participating in WhatsApp chats, or using ringtones in Ghana have proven effective for promoting blood donation [16]. In this sense, the emergence and growing use of digital collaborative platforms means that they could become a very useful tool for encouraging blood donation, especially among younger people. An example of this is Blooders.org, a platform that not only educates about blood donation, but also promotes and facilitates it via a digital community. The aim is to improve the experience of blood donation in general, and to encourage blood donation in emergency and natural catastrophe situations in particular, as was demonstrated very well during the earthquake in Mexico on 9 September 2017 [13].

The aim of this work is to study, for the very first time, the motives for blood donation via digital platforms and, with this knowledge, to develop strategies to promote the use of this new digital tool. Furthermore, and taking into consideration not only the growing need for transfusion but also the mobility limitations imposed during the COVID-19 pandemic, we have situated our study within the context of the pandemic. It is important to note that our research goal is to provide initial preliminary evidence. Consequently, the effects of the pandemic on blood donation, the ways in which the use of digital platforms affects blood donation, and the comparison of the motives of donors that use/do not use digital platforms are not part of our investigation. Our aim is much more modest: to provide initial evidence of the motives underlying the use of digital platforms for blood donation. We believe that this initial evidence makes a new contribution to the literature for a number of reasons. First, this study provides analysis of a novel mobilisation and donation tool that, moreover, has very much proven its usefulness in other types of donation. Second, we recognize the need to study the use of new recruitment and donation strategies as a result of the limitations imposed during the COVID-19 pandemic. However, we have no specific information to compare blood donation motivation during the COVID-19 pandemic to other situations. Therefore, the COVID-19 pandemic is not a core component of this study and, as such, is purely contextual. 

## 2. Theoretical Background and Hypotheses

### 2.1. Motivations for Blood Donation

Various studies have analysed the factors that motivate donors to give blood [17,18,19,20]. Research has put their motives into three main groups: altruism, self-interest, and response to direct or social appeal [21]. Altruism is considered to be the main motive in the majority of the studies, and it is based on the desire to help others by donating without receiving anything in return [22,23]. Self-interest motives are based on the pursuit of some kind of individual interest, such as getting satisfaction from helping others or being rewarded for donations [24,25]. Lastly, response to direct or social appeal motives are extrinsic ones originating from marketing campaigns run by blood collection institutions, or from the influence exerted by reference groups [21,26]. 

From the three groups of motives mentioned above, the literature has gradually evolved in terms of understanding the multidimensional set of motives affecting blood donation. Altruism has been linked to an awareness of need, a predisposition towards blood donation, social pressure, and attachment and family relationships in the explanation of blood donation [27]. Other studies have described a variety of intrinsic motives, such as goodwill, gratification, self-esteem and recognition [25]. Interestingly, the latter factors are the most strongly associated with donor retention [27]. This is especially important because it allows efforts to be focused on intrinsic motives when it comes to the development of effective strategies to increase donor commitment. Research has also made progress with regard to the identification of factors which have a negative influence on donation. Among these are fear, lack of care by healthcare staff, adverse physical reactions, the length of the process, a bad experience, and not having a suitable place for blood donation [28]. 

Lastly, the most recent studies have again expanded on both the motivational interests and the agents involved in donation. Within this context, the importance of anticipated emotions [29] has been noted, as has the market-oriented tendency of the bodies managing donation [30]. Regarding the agents, the need to distinguish and characterise the recruitment and engagement motives and strategies for non-donors [31], occasional donors and active donors has also been highlighted [32]. In this respect, intrinsic and extrinsic stimuli have been found to be important for motivating non-donors [33]. 

### 2.2. Participation in Collaborative Digital Platforms

The advent of digital platforms (e.g., Uber, Airbnb and Blooders.org) has profoundly transformed both economic activity and exchanges between people who have no commercial intention. Within this context, field research has pointed out the collaborative nature of this new set of online resource circulation systems, which enable people to both obtain and provide, temporarily or permanently, valuable resources or services through direct interaction with other people or through a mediator. A provider is a person who provides a specific resource or service, either directly to an obtainer or indirectly through a mediator. Thus, collaborative provision refers to re-exchange or reuse, such as reselling or second-hand purchase, subleasing, swapping, free or paid donation, and reconditioning or refurbishing [34]. Following this approach, we understand collaborative blood donation as blood donation between a person and a healthcare institution mediated by a digital platform (see Supplementary File S1).

Participation in collaborative platforms is based on a multidimensional set of motives [35,36]. The first motive is people’s ability to know about and operate within digital platforms, which includes knowledge about the existence and utilities of the platforms [37,38,39]. Secondly, research on collaborative exchanges has also confirmed that the antecedents of digital participation might be different depending on whether people are acting as obtainers or providers [40,41]. In the literature, strong support has been found for the prevalence of utilitarian motives, especially economic and practical ones, at the obtainment stage [42,43]. However, a broader set of non-utilitarian and pro-social drivers, such as the creation of better communities through alternative non-profit exchanges, sustainability, solidarity, or helping people, also foster provision [44,45]. Third, research has also found a set of barriers hindering participation in collaborative exchanges [35,36]. Procedural, process and privacy risk concerns, distrust among participants, and effort expectancies are the most common barriers identified in the literature [46,47]. 

### 2.3. Theory of Planned Behaviour and Participation in Collaborative Platforms

Participation in collaborative platforms was initially formulated as an acceptance intention, and therefore the Technology Acceptance Model (TAM) approach could be taken. In TAMs, technology acceptance is considered a determinant of technology use [48,49]. Thus, the acceptance of digital platforms can also be considered a determinant of their use. While such models have been widely employed to explain the uses of many different technologies, a number of problems have been highlighted [50]. Primarily, the overly generic nature of TAMs has been underscored, as have the difficulties associated with correctly predicting various alternative uses of a particular technology, and the lack of any link to certain psychological or social constructs that predict behaviour, such as attitudes or intentions [51]. In addition, TAMs are clearly associated with the analysis of the use of a particular technology, rather than a service or platform that—as is the case with digital platforms—has an obvious technological basis and, at the same time, other non-technological dimensions [52]. In this respect, motivational research has taken the acceptance of a platform as a dimension of individual behaviour, and has employed theories and models from the spheres of psychology and social psychology [53].

In the literature on collaborative exchanges, the use of the Theory of Planned Behaviour (TPB) as an alternative way of modelling the acceptance of digital platforms or services has been proposed [54]. TPB models postulate that the individual intention of carrying out a behaviour is a predictor of an individual’s future behaviour [55,56]. The intention to carry out a certain type of behaviour is linked to the attitude towards that behaviour, to the subjective norm, and to perceived control over that behaviour. In the specific case of participation in digital collaborative platforms: (1) the attitude towards participation reflects the extent to which a person believes that his or her participation will help him or her achieve the desired objectives, (2) the subjective norm is associated with a person’s perception of the social pressure on him or her to participate or not in collaborative platforms, and (3) perceived control over participation in digital P2P platforms refers to the ease or difficulty of participating in such platforms. 

The use of a TPB model has several advantages. First, owing to its broad level of abstraction, this model can be used to predict any type of behaviour, including participation in digital collaborative platforms. Second, due to its principle of aggregation, the TPB model allows for the incorporation of multiple behaviours, especially for the joint consideration of obtainers and providers [57]. Third, the reflective nature of the TPB model enables systematic, integral constructs of behaviour to be built, which would anticipate the intention to collaborate. Thus, an evaluation of the structure of beliefs and intentions to collaborate by using TPB models can help us to identify constructs that are more specialised, sophisticated and multidimensional [58].

**Figure 1:**
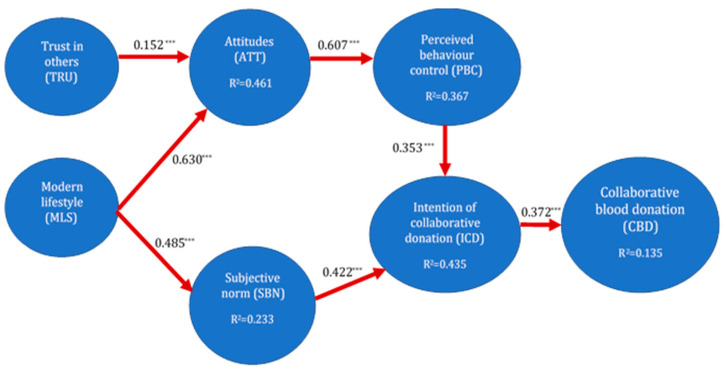
Structural model (direct effects) of collaborative blood donation (CBD). Note: *** *p* = 0.001.

In short, and following the postulates of the TPB, in this study we will refer to behaviours in the sense of digital platform use behaviours. We will also refer to motives as antecedents that predict the behaviour of the use of digital platforms for blood donation. Among these motives, we will find intrinsic ones such as trust, attitudes and behavioural control, and extrinsic motives such as the subjective norm. The model to be contrasted is shown in Figure 1: trust and modern lifestyle motives, and the hypothesised relationships between them and the TPB dimensions (attitudes, behavioural control and the subjective norm), and between the intention to donate and actual blood donation via digital platforms. 

### 2.4. Hypotheses

Research on collaborative exchanges has frequently highlighted the fact that trust between users is important to explaining collaborative attitudes [47,59,60]. What we understand by trust in others is the belief that people participating in an exchange will act in a socially responsible way and fulfil the ethical expectations of their behaviour without cheating, or harming or exploiting other users [61,62]. In environments of high uncertainty, such as digital platforms, a lack of trust among participants can clearly deter collaborative attitudes [63,64]. Conversely, by reducing uncertainties, trust allows psychological behavioural barriers to be overcome, thus acting as a driver of positive attitudes [60,65]. Another important predictor of positive attitudes and the social recognition of collaboration is a modern lifestyle. Participation in digital platforms has been linked to modernity through the characterisation of obtainers and providers (urban, educated young people with digital skills) and the contemporaneity, identity and independence that a lifestyle featuring collaborative exchanges entails [66,67].

Following the postulates of the TPB, trust in others and the modern lifestyle can be associated with attitudes and behavioural control to generate collaborative blood donation (intention and effective donation). This link would have to do with altruism. Trust in others, reinforced with appropriate attitudes and behavioral control, can motivate altruistic contributions to the community [22,68]. In this context, the first research hypothesis is established:

**H1.**  Trust is linked to attitudes and behavior control to predict blood donation (intention and effective donation).

However, it is also possible to relate this link to individual motivations. A modern lifestyle, reinforced with the necessary attitudes and behavioural control, can motivate greater individual satisfactions of helping others [69]. Thus, a second working hypothesis can be postulated:

**H2.**  The modern lifestyle is linked to attitudes and behavioural control to predict blood donation (intention and effective donation).

Finally, it is possible to postulate another predictive path that links the modern lifestyle with the subjective norm in the development of collaborative blood donation (intention and effective donation). This link is related to the motives of direct and social attraction [24]. Belonging to the collective of collaborative blood donors creates a sense of pride that can be fueled by a modern lifestyle and the satisfaction of belonging to a community. Thus, the third working hypothesis is:

**H3.**  The modern lifestyle is linked to the subjective norm to predict blood donation (intention and effective donation).

## 3. Materials and Methods

### 3.1. Study Design

Along the lines of TPB-based and applied research into participation in digital platforms for commercial purposes, a motivational model adapted to blood donation via digital platforms was designed and tested [35,36,38,40,70]. To that end, a set of motivational factors predicting the attitude, subjective norm and perceived behavioural control constructs was identified. These drivers and barriers encompassed a considerable number of predictive technological, utilitarian, pro-social, and environmental dimensions. They included economic benefits; uniqueness; variety; ubiquity and availability; social experience; problems related to risk, privacy and scarcity; the prestige and independence of ownership; ecological sustainability; anti-capitalism; a sense of belonging to a community; a modern lifestyle; effort expectancy; and trust in others. After testing all of these motivational factors to predict the attitude, subjective norm and perceived behavioural control, the model was completed with the predictive interaction between these three constructs and behavioural intention and donation behaviour (the aim, model, items and constructs analysed in the study can be found in Supplementary File S1).

An ad-hoc study was designed to assess the motives of collaborative blood donors. As a result of the lack of reference research on Collaborative Blood Donation (CBD), a conceptual analysis framework was adapted, from which a multidimensional set of drivers and barriers was derived. Of the 16 initially proposed motivational constructs, robust predictive capacity was only obtained for two: modern lifestyle and trust in others. The CBD model obtained is shown in Figure 1. According to these results, trust and modern lifestyle motives serve as independent constructs, and the relationships between them and the TPB dimensions (attitudes, behavioural control and subjective norm) and outputs (the intention to donate and actual blood donation via digital platforms) are contrasted. 

Based on TPB modelling, a survey was developed to enable us to obtain primary microdata. The research model was tested using these microdata. This study complies with the ethical criteria of social research that the Open University of Catalonia (UOC, Barcelona, Spain) [71] and the Austral University of Chile (UACh, Valdivia, Chile) have set out for their researchers. Furthermore, as in other eHealth research carried out by this research team [72,73], the study followed the Checklist for Reporting Results of Internet E-Surveys (CHERRIES) guidelines [74].

### 3.2. Participants

We recruited participants from our Latin-American and Spanish student pools, and from our academic contacts in social, professional and research networks at UOC and UACh. Non-probabilistic sampling, also known as random accidental sampling, was used to obtain the sample [75]. The participants did not receive any monetary or non-monetary rewards. In this study, no specially protected data were collected, and there was no reference to ideology, religion or beliefs. In addition, in order to ensure the confidentiality of the results obtained, the questionnaires were anonymous, meaning that the participants could not be identified in any way whatsoever. We invited a total of 990 participants to take part in the survey via e-mail and postings on social media. A bi-weekly reminder was sent to non-participants during the fieldwork period. The survey was designed and prepared to be completed online as a self-report instrument. The respondents took approximately 15 min to complete the survey. The fieldwork was carried out between 17 February and 17 May 2020.

In total, 389 participants started the survey, and 325 completed it. Given the exceptional circumstances of lockdown as a result of the COVID-19 pandemic, as well as the extent and breadth of the motives discussed, the response (39.2%) and completion (83.5%) rates can be considered high. Despite such active participation, it is important to mention the preliminary nature of the research. Because there is no representative sample, the results obtained are only preliminary and must be taken with caution. The cross-tabs and ANOVA analyses comparing participants’ and non-participants’ sociodemographic characteristics did not indicate any significant non-response bias. In order to ensure the data quality, the participants were excluded if they did not provide most of the information requested, or if they did not answer the care and understanding questions successfully. This resulted in a final sample of 302 valid participants. Descriptive information on the health status, blood donation, digital skills and sociodemographic characteristics of the participants is provided in Supplementary File S2.

Regarding gender, 54.7% of the sample was male. The participants’ mean age was 51.7 years (SD = 1.08), distributed across the following categories: under 30 years old (8.2%), 30–39 years old (14.9%), 40–49 years old (18.2%), 50–59 years old (24.3%), 60–69 years old (19.9%), and 70 years old or more (14.5%). Regarding household status, 28.8% of the participants lived with a partner and had children, 21.3% lived alone and had children, and 24.5% lived with a partner and did not have children. Most of the participants resided in small or medium-sized towns/cities (54.9%), and nearly one third resided in large towns/cities (31.0%). In relation to their country of origin, the sample of participants included citizens from 11 Latin American countries and Spain. Worthy of note is that there were 189 participants from Chile (62.6%), 34 from Spain (11.2%), 30 from Colombia (9.9%) and 18 from Puerto Rico (6.0%). The rest of the participants (10.3%) were distributed in small country samples. Regarding their professional situation, most of the participants were qualified professionals (47.5%) working in education and research (27.5%), health and well-being (18.0%), public administration (17.3%), and professional and business services (11.6%). The participants were highly educated. Most of them had completed at least a bachelor’s degree (77.1%). Compared to the official data provided by the Spanish and Latin American Statistical Offices (Labour Force Surveys), our sample was biased towards highly educated people. 

Regarding health status, 75.2% of the participants said they were in good or very good health, 58.9% did not have any chronic illness or disease, and 54.0% of the respondents had someone close to them with a chronic illness or serious health problem. Half of the participants had donated blood at some stage, although 42.5% of the donors had only donated blood once in the past five years. Among the donors, the donation experience was rated mostly good or very good (80.8%), and the main reasons for donation (multiple responses) were personal awareness (62.2%), helping others (49.5%), and in response to a specific donation campaign (29.3%). However, 69.5% of the donors believed that the blood donation process could be improved through better use of digital technologies and platforms. Among the non-donors, the main reasons for not donating were (multiple responses) fear of complications after donation (21.3%) and needles (20.5%). Finally, it is important to note the respondents’ high levels of digital technology skills and use. Daily, 85.2% used the Internet, 72.2% sent and received e-mails, 70.4% participated in social media and chats, and nearly a quarter used digital collaborative platforms. The digital technology use in the health field was also remarkable. Daily, 30.6% of those surveyed used the Internet, 18.0% sent and received e-mails, 13.7% participated in social media, and around 10% did so in digital health platforms.

### 3.3. Measures

The constructs were adapted in accordance with the steps recommended in the literature [76,77]. First, the items were translated from English to Spanish by research experts (university lecturers), and by language experts belonging to the Language Service of UOC and UACh. Second, we created a focus group to discuss the translated items (e.g., equivalence of meaning). Third, language experts back-translated the items from Spanish to English. Finally, we checked the equivalence of meaning between the original and adapted versions. 

The compared model began with two motivational predictors: trust in other users (TRU) and modern lifestyle (MLS). The TRU construct was tested by means of two items, e.g., “Other CBD users are trustworthy”. The MLS construct was captured by means of two items, e.g., “To me, CBD represents an up-to-date lifestyle”. After the two motivational predictors, the tested model incorporated relationships between the four basic constructs of the TPB models: attitudes (ATT), subjective norms (SBN), perceived behavioural control (PBC), and the intention of collaborative donation (ICD). Finally, behavioural intention predicted collaborative blood donation (CBD). Some examples of the items used in these constructs are: attitude (ATT, three items), “Using CBD is a good idea”; subjective norm (SBN, two items), “People who are important to me think that I should participate in CBD”; perceived behavioural control (PBC, two items), “Using CBD is entirely within my control”; intention of collaborative donation (ICD, two items), “I intend to use CBD in the future”; and collaborative blood donation (CBD, two items), “I have experience with CBD”. We used a 5-point Likert scale in all of the constructs analysed: the response range was from 1 (strongly disagree) to 5 (strongly agree). All of the constructs analysed were reflective. The origin, bibliographic references, items and descriptive statistics of the constructs analysed are shown in Tables S1.3 and S3.1 of Supplementary Files S1 and S3 of the Supplementary Materials. 

### 3.4. Statistical Analysis

The data were analysed using Partial Least Square-Structural Equation Modelling (PLS-SEM), a variance-based structural equation modelling approach [78]. Within some contexts of analysis, this statistical methodology is, for various reasons, preferable to other covariance-based methods such as SEM [79]. First, PLS-SEM shows accurate estimates of the paths among constructs by analysing the measurement and structural models simultaneously [80]. Second, PLS-SEM is an appropriate statistical method for exploratory or predictive studies that test moderation effects and analyse complicated relationships [81]. Third, in recent years, PLS-SEM has been used in a growing number of knowledge areas, especially Internet-based research [35,82]. However, despite its potential, the use of PLS-SEM in health-related research is still very low. Only studies on tourism and medical hospitality have based research on this methodology [83,84]. The model was tested using 3.2.9 SmartPLS.3 software (SmartPLS GmbH, Bönningstedt, Germany).

## 4. Results

### 4.1. Measurement Model

Internal consistency (construct reliability), and convergent and divergent validity were examined in order to assess the reflective measurement model. The skewness and kurtosis values (reported in Supplementary File S3) suggest that the variables can be assumed to be normally distributed (below the threshold of 2.58). Multicollinearity diagnoses were addressed by testing the variance inflation factor (VIF) among the constructs. Given that all of these values were below the threshold VIF = 10, multicollinearity might not be a concern (Supplementary File S3). Additionally, composite reliability was used to evaluate the internal consistency. Table 1 shows that the composite reliability scores were higher than 0.7, indicating internal consistency reliability.

Meanwhile, the convergent validity was evaluated by considering the outer loadings of the indicators and the average variance extracted (AVE) [79,85]. The standardised outer loadings of the reflective indicators were higher than 0.7, indicating that the shared variance between the constructs and their respective indicators was larger than the measurement error variance. Furthermore, the AVE value of all of the constructs was higher than 0.5, reflecting that, on average, the construct explained more than half of the variance of its indicators.

Finally, the discriminant validity was assessed in accordance with the Fornell–Larcker and the Heterotrait–Monotrait (HTMT) criteria [79,85]. The data presented in the correlation table show that the square root of all of the AVE constructs was higher than the correlations between the study constructs, thus fulfilling the Fornell–Larcker criterion (Table 2). In addition, the model reflects a good fit according to the HTMT ratio, as the Heterotrait correlations were smaller than the Monotrait ones. Table 2 shows that the HTMT ratios were smaller than 0.9. Moreover, bootstrapping analysis, based on 500 samples, showed that the HTMT values were significantly different from 1. These results indicate that all of the constructs were empirically distinct.

### 4.2. Structural Model

The structural model results were assessed in accordance with the systematic approach recommended in the literature on PLS-SEM [78,81]. Firstly, all of the VIF values were lower than 5, suggesting the absence of multicollinearity. Secondly, bootstrapping based on 500 subsamples was applied in order to assess the statistical significance of the path coefficients. The direct effects were evaluated by applying a one-tailed test for a Student’s t-distribution (95% confidence interval). As reflected in Table 3, the direct effects considered in the model were significant at a maximum 0.001 level (t ≥ 3.092), meaning that all of the coefficients were significantly different from 0. All of the signs were positive, and the confidence intervals excluded 0 in all cases. Thirdly, the magnitude of the coefficients of determination indicated that the model had adequate predictive power for all of the exogenous variables [79,85]. The adjusted-R2 values indicated moderate explanatory power. Attitudes and the intention of collaborative donation had the highest explained variances (0.461 and 0.435, respectively). Fourthly, the effect size was evaluated using Cohen’s f2, which was above the base level of 0.02 in all cases, indicating that the study variables represented key antecedent constructs of their respective exogenous variables. Finally, the cross-validated redundancy index (Q2) was used to assess the predictive relevance of the model. The results summarised in Table 3 and Figure 1 confirm that the structural model had satisfactory predictive relevance for all of the endogenous constructs, as Q2 was higher than 0 in all cases.

The results reflected in Table 3 indicate that the paths from trust in others and modern lifestyle to collaborative blood donation were significant and positive. However, our model hypothesized various pathways through the indirect effects established by the latent intermediate variables: attitudes, subjective norms, perceived behavioural control and intention of collaborative donation. Bootstrapping based on 500 subsamples was again used to test the statistical significance of specific indirect effects, and to generate t-statistics and *p*-values. The results obtained show that, of the three indirect ways established to predict collaborative blood donation (see the indirect effects in bold in Table 4), the one connecting a modern lifestyle with the subjective norm and intention of collaborative donation was the most relevant to explain collaborative behaviour. The path starting from modern lifestyle and connecting with attitudes, behavioural control and intention of collaborative donation was also significant. Finally, the effect value of the indirect path starting from trust in others was markedly lower than the two previous indirect paths (see Figure 1).

## 5. Discussion

### 5.1. Theoretical Implications

The aim of this study was to evaluate people’s motives for blood donation using digital platforms. It was an exploratory and predictive study that examined a measurement model and a structural model of the antecedents and behaviour of digital collaborative communities for blood donation.

The TPB is one of the most robust conceptual frameworks for the study of human behaviour [55]. This theory has already been widely used in research on blood donation behaviour precisely because of the voluntary nature of the act of donating blood, in accordance with the behavioural control function [3,86,87,88,89,90]. The predictive capacity of this model has gradually been improved by including additional predictors [17,90,91,92,93]. Theoretically justified, the aim of these new predictors is to improve the delineation of the parameters of blood donation behaviour and to capture a significant portion of variance [94,95,96,97]. 

Research has extended the use of the TPB from behavioural intention to actual donation behaviour within a real blood donation context, concluding that the TPB was more effective for predicting blood donation intentions than it was for predicting blood donation behaviour [98]. Similarly, research into the area of technology has often used the TPB for the purpose of explaining a multidimensional set of motives for collaborative platform use [35]. 

At the intersection between research into the motives for blood donation and for participation in collaborative platforms, our study represents the very first time that the TPB model has been used within the context of collaborative blood donation. In relation to the participants in collaborative blood donation, TPB modelling allowed us to separate out their donation attitudes, intentions and actual behaviour [38]. In general, attitude was considered the main determinant of behavioural intention. However, because of the unique nature of the shared resource, the compared model interposed perceived behavioural control as a mediating construct between attitude and behavioural intention. It was hypothesised that the explanation for such mediation would be linked to the need for collaborative blood donors to perceive that they controlled donation behaviour, because they were donating a resource that was very valuable to them [93]. Likewise, the social pressure (subjective norm) exerted by the collaborative blood donor’s environment would also foster the intention to collaborate. Based on the TPB model, it was to be expected that the subjective norm would be a predictor of the intention of collaborative blood donation when an individual’s behaviour was largely motivated by social factors, such as the influence that other members of a community might have on him or her [59,99], especially in pandemic situations [100]. Lastly, through the link between intention and action, the intention to donate blood would be a predictor of actual collaborative blood donation [56].

### 5.2. Management and Social Implications

Despite having tested 16 motivational constructs, identified in the literature as predictors of digital platform use in exchanges of goods and services, our analysis showed that only trust in others and modern lifestyles had predictive power. Trust in others enables the psychological barriers to collaboration to be overcome, and it is a lever for triggering positive attitudes [60,65]. Modern lifestyles, which are generally characteristic of urban, educated young people with digital skills, are associated with a contemporary and independent identity, and the use of collaborative platforms to exchange all kinds of resources [66].

Following the paths resulting from the model, trust in others and modern lifestyles were associated with attitudes and behavioural control for the generation of the intention to donate and actual donation. These predictive paths are linked to altruism [68]. Besides contributing to the community, altruistic collaborative donors may develop attitudes and controlled behaviours that end up fostering a useful action that could be extrapolated to others [22]. However, trust in others and modern lifestyles could also be related to individual interest motives [69]. The satisfaction or merit from helping others fitted well with the attitudes and behavioural control path in the generation of intentions and actual donations. Meanwhile, our study also obtained another path linking modern lifestyle to the subjective norm in the development of intentions to donate blood, and of collaborative blood donation. This predictive path is linked to the response to direct and/or social appeal motives. In this motivational path, the recognition that collaborative blood donors achieve is connected with a sense of pride and belonging to a group that is valued by society. The act of joining this group is driven by the value (intrinsic and extrinsic) placed on a certain collaborative lifestyle, which would motivate the intention to donate blood and actual blood donation [24].

Research has also shown that blood donation does not solely respond to stimuli linked to positive emotions. It can also be promoted by avoiding negative emotions [101], especially those linked to non-donation, fear [102], guilt, or anxiety [103]. Within this context, the importance of anticipated emotions has recently been noted as a major predictor of blood donation [104,105]. Such anticipated emotions, which can reflect both positive and negative mood states in relation to the future donation of blood, may explain both the intention and the non-intention to donate. This is particularly so in the case of negative mood states [106]. Within collaborative donation, which is strongly mediatised because of the digitisation of blood exchange, our results suggest that anticipated emotions linked to trust among donors and a positive mood state associated with a modern lifestyle are relevant when it comes to triggering the predictive paths of the drivers and barriers to collaborative blood donation.

### 5.3. Limitations

Our findings should be interpreted in light of several limitations. First, the sample data were obtained from people living in Spain and Latin American countries. Among the latter, there were considerable differences in the degree of implantation of digital platforms and the spread of COVID-19. Second, it is also worth noting that our study addressed the motives for collaborative blood donation without looking at the study participants’ previous roles as non-occasional or regular donors. Thirdly, the preliminary nature of the research should be pointed out. The resulting motivational pathways were obtained from a sample that is not representative at the populational level, either of blood donors or digital platform users. The lack of representativeness makes it necessary to take the motivational paths obtained with caution, and to expand the analysis samples in future research. In fact, much more attention will undoubtedly need to be paid to cultural and health diversity, and to previous involvement or otherwise in blood donation. Despite these limitations, this is the first known study to conduct research into blood donation via collaborative platforms within the context of a disease outbreak. The results from our study provide the first insights into the motivational paths that people follow for collaborative blood donation during the COVID-19 pandemic context.

## 6. Conclusions

By determining the paths underpinning collaborative blood donors’ motives, the results of this study provide strong support for the usefulness of the TPB model within the context of digital platform use and blood donation. As such, these paths represent one of the very first attempts to predict intentions of donation and collaborative donation by taking a PLS-SEM approach. 

Our study obtained three main prediction paths. The first two started from feelings of trust in the digital community and a positive mood state associated with a modern lifestyle, and they were linked to attitudes and behavioural control in the explanation of the intention to donate and actual blood donation. The third path started from the modern lifestyle, and was linked to the subjective norm in the prediction of intention and actual donation. The first two paths were associated with altruistic motives and self-interest, whereas the third was linked to the response to direct or social appeal motives. In any case, the paths obtained started from anticipated emotions and drew specific behaviour trajectories within the contexts of blood donation and digital platforms. 

These three motivational paths obtained from our study could be used in the recruitment of future donors via digital collaborative platforms, which are widely used in other areas of digital life. Our results may also be useful for the formulation of strategies to ensure that donations continue to be made in a manner that is transparent (P2P) for both blood donors and receivers [95,107].

## Figures and Tables

**Table 1:** Summary of the measurement model.

Latent Variable	Indicators	Internal Consistency Reliability	Convergent Validity		Discriminant Validity
		Composite Reliability >0.7	Loadings >0.7	AVE >0.5	HTMT Confidence Interval Does Not Include 1
TRU	TRU1	0.946	0.958	0.897	Yes
	TRU3		0.936		
MLS	MLS1	0.917	0.920	0.847	Yes
	MLS3		0.921		
ATT	ATT1	0.913	0.915	0.778	Yes
	ATT2		0.921		
	ATT3		0.806		
SBN	SBN1	0.935	0.940	0.877	Yes
	SBN3		0.934		
PBC	PBC1	0.904	0.908	0.825	Yes
	PBC2		0.909		
ICD	ICD1	0.909	0.896	0.833	Yes
	ICD2		0.930		
CBD	CBD1	0.945	0.950	0.896	Yes
	CBD2		0.943		

**Table 2:** Discriminant validity.

**Fornell-Larcker Criterion**							
	**ATT**	**CBD**	**ICD**	**MLS**	**PBC**	**SBN**	**TRU**
**ATT**	0.882						
**CBD**	0.320	0.947					
**ICD**	0.641	0.372	0.913				
**MLS**	0.665	0.213	0.576	0.920			
**PBC**	0.607	0.258	0.545	0.604	0.908		
**SBN**	0.522	0.371	0.583	0.485	0.454	0.937	
**TRU**	0.298	0.209	0.220	0.232	0.255	0.267	0.947
**Heterotrait-Monotrait Ratio (HTMT)**							
	**ATT**	**CBD**	**ICD**	**MLS**	**PBC**	**SBN**	**TRU**
**ATT**							
**CBD**	0.370 [0.265;0.481]						
**ICD**	0.779 [0.681;0.893]	0.435 [0.338;0.529]					
**MLS**	0.793 [0.701;0.913]	0.250 [0.147;0.361]	0.715 [0.602;0.821]				
**PBC**	0.742 [0.634;0.853]	0.308 [0.203;0.413]	0.685 [0.581;0.792]	0.752 [0.646;0.864]			
**SBN**	0.611 [0.504;0.722]	0.425 [0.312;0.524]	0.694 [0.578;0.802]	0.578 [0.470;0.682]	0.552 [0.451;0.670]		
**TRU**	0.342 [0.255;0.421]	0.231 [0.128;0.314]	0.250 [0.143;0.261]	0.268 [0.152;0.370]	0.302 [0.112;0.405]	0.302 [0.100;0.407]	

Notes: Diagonal (bold) items represent the square root of the AVE. The elements below the diagonal are correlations between constructs. The HTMT ratio with BC 95% confidence intervals was based on 500 subsamples.

**Table 3:** Summary of the direct effects.

Endogenous Variable Structural Path	Direct Effect	*t*-Value	Bootstrap 95% CI ^1^	Cohen’s f2
Attitudes (ATT) TRU ➔ ATT MLS ➔ ATT (R2 = 0.461; Q2 = 0.453)	0.152 0.630	2.691 *** 12.343 ***	[0.065;0.254 [0.536;0.704]	0.041 0.701
Subjective norm (SBN) MLS ➔ SBN (R2 = 0.233; Q2 = 0.227)	0.485	7.302 ***	[0.375; 0.590]	0.308
Perceived behavioural control (PBC) ATT➔ PBC (R2 = 0.367; Q2 = 0.332)	0.607	13.124 ***	[0.529; 0.681]	0.585
Intention of collaborative donation (ICD) PBC ➔ ICD SBN ➔ ICD (R2 = 0.435; Q2 = 0.276)	0.353 0.422	5.590 *** 7.053 ***	[0.254;0.461] [0.319;0.517]	0.176 0.252
Collaborative blood donation (CBD) ICD ➔ CBD (R2 = 0.135; Q2 = 0.039)	0.372	7.561 ***	[0.289; 0.448]	0.160

Notes: *** *p* < 0.001. ^1^ All intervals are significant.

**Table 4:** Summary of the indirect effects.

Latent Variable	Indirect Effect	*t*-Value	Bootstrap 95% CI ^1^	
			5%	95%
**MLS -> ATT -> PBC -> ICD -> CBD**	0.050 ***	3.039	0.028	0.080
PBC -> ICD -> CBD	0.131 ***	4.178	0.085	0.184
ATT -> PBC -> ICD -> CBD	0.080 ***	3.556	0.048	0.118
**TRU -> ATT -> PBC -> ICD -> CBD**	0.012 **	2.196	0.005	0.022
SBN -> ICD -> CBD	0.157 ***	4.763	0.103	0.212
**MLS -> SBN -> ICD -> CBD**	0.076 ***	3.293	0.043	0.102
MLS -> ATT -> PBC -> ICD	0.135 ***	4.009	0.084	0.195
ATT -> PBC -> ICD	0.214 ***	4.820	0.145	0.287
TRU -> ATT -> PBC -> ICD	0.033 **	2.221	0.012	0.059
MLS -> SBN -> ICD	0.205 ***	4.237	0.131	0.289
MLS -> ATT -> PBC	0.383 ***	6.510	0.281	0.480
TRU -> ATT -> PBC	0.092 **	2.629	0.035	0.150

Notes: *** *p* < 0.001; ** *p* < 0.05. ^1^ All intervals are significant. In bold, hypothesized indirect effects.

## Data Availability

No additional data available.

## Supplementary Materials

The following are available online at https://www.mdpi.com/article/10.3390/ijerph18084270/s1. Supplementary File S1: Collaborative Blood Donation (CBD) Survey. Supplementary File S2: Characteristics of the participants. Supplementary File S3: Measure items—descriptive statistics.
