# Supplementary material for: Using Digital Platforms to Promote Blood Donation: Motivational and Preliminary Evidence from Latin America and Spain
Source: Int J Environ Res Public Health. 2021 Apr 17;18(8):4270. doi: 10.3390/ijerph18084270 (PMC8073325; doi:10.3390/ijerph18084270)
Supplement: Supplementary file 1 [file ijerph-18-04270-s001.zip › Supplementary File S1.pdf]

Supplementary File S1. Collaborative Blood Donation (CBD) Survey

Introduction

In the literature on collaborative exchanges, the use of the Theory of Planned Behaviour (TPB) as a way of modelling the acceptance of digital platforms or services has been proposed. TPB models postulate that the individual intention of carrying out a behaviour is a predictor of an individual's future behaviour. The intention to carry out a certain type of behaviour, e.g., participation in blood donation activities via digital platforms, is linked to the attitude towards that behaviour, to the subjective norm and to perceived control over that behaviour.

Attitude towards participation reflects the extent to which a person believes that his or her participation will help him or her achieve the desired objectives. Subjective norm is associated with a person's perception of the social pressure on him or her to participate or not in collaborative platforms. Perceived control over participation in digital P2P platforms refers to the ease or difficulty of participating in such platforms. In short, we have adapted and tested a TPB model that brings together a multidimensional set of predictive factors of the intention to donate and actual blood donation via digital platforms. Figure 1 shows the initial TPB theoretical model that has been tested.

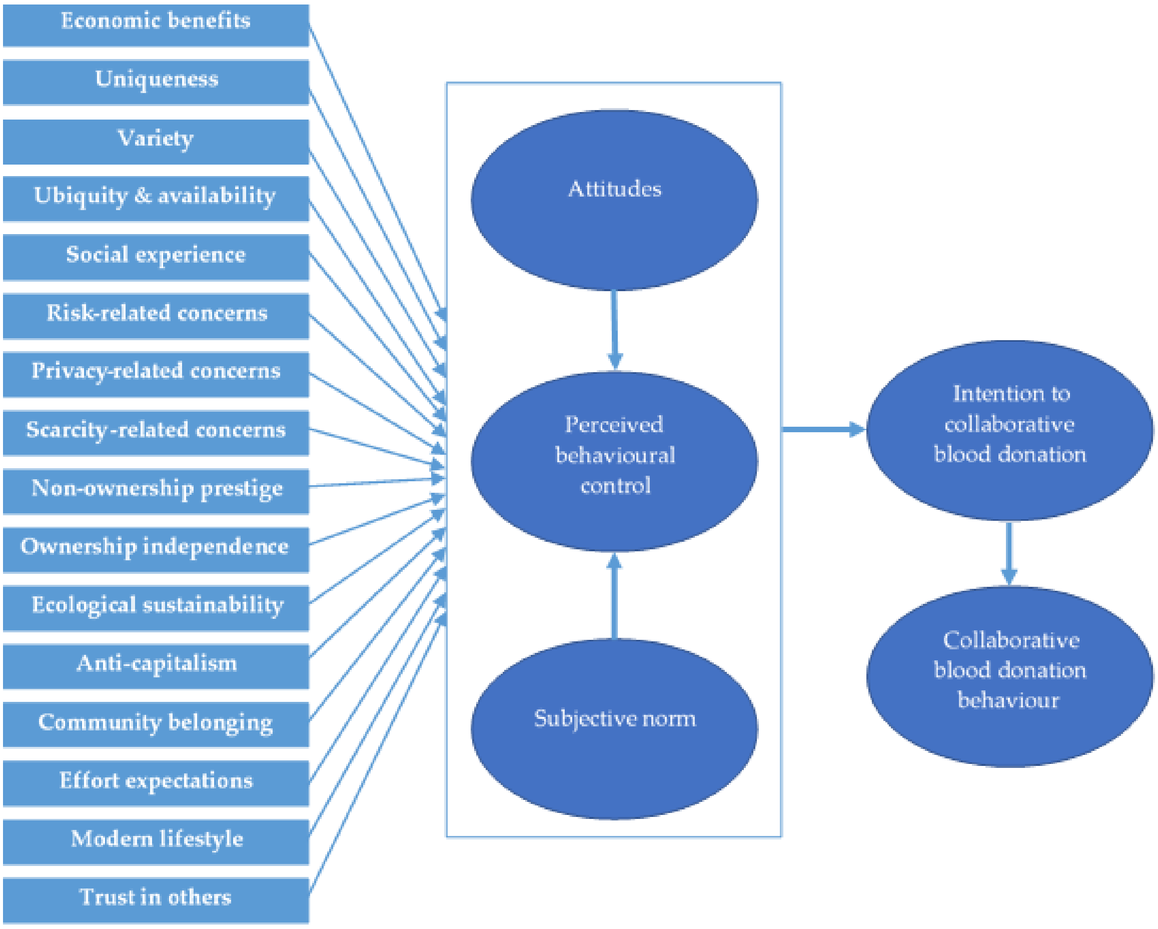

Figure 1. Model of Collaborative blood donation (CBS)

Research aims

Welcome and thank you very much for participating in the Survey. Only, we will take 15-20 minutes of your time.

The survey deals with the use of collaborative platforms for blood donation. First of all, we would like to explain our approach to the end of collaborative platforms. You should bear in

mind that, for your participation, it is not important that you have experience in using these platforms. Your opinion interests us in any case.

Collaborative platforms are explained and understood in various ways in the media, so that it is often not entirely clear what this term refers to. In this survey we will focus on a clearly defined aspect, blood donation between a person and a healthcare institution mediated by an online platform. We will summarize this action as "Collaborative Blood Donation" and use the abbreviation CBD. Despite the fact that blood donation is a pro-social act in itself, that is, an act where people donate their blood to help other people, in this survey we want to study whether the use of collaborative platforms modifies motivations and barriers to donation (or even the amount of blood donated) compared to more traditional donation campaigns / technologies (press, radio, TV, bus blood donation, etc).

To clarify what exactly we mean by CBD, consider the following criteria (we also provide negative examples of each criterion):

- The transactions operate on a donation basis; therefore, they comprise property transactions and rights to use blood without monetary or other remuneration. Despite the fact that in many countries, paying to donate blood is prohibited, DCS does not contemplate illicit donations. Regarding returns, only those allowed for health reasons (for example, a snack or a drink after donation) are included.
- Transactions take place between donors (individuals) and recipients (generally national or international health entities, such as the Red Cross or Red Moon). CBD does not contemplate donations between individuals, or between individuals and for-profit firms/entities, without the participation of a healthcare entity. This entity and the health professionals who participate in the donation are in charge of ensuring that the donation is carried out in healthy terms for the donor.
- Generally, donations are organized through campaigns promoted by public agencies (governments or ministries of health, WHO) or health entities (hospitals, assistance centers, international entities). Provided both of the above requirements are met, CBD also provides for regular blood donation without any organized campaign.

Some of the survey questions point to her experience with CBE. If you are inexperienced, answer the question from a hypothetical or general point of view. Answer all questions in the most honest and intuitive way possible. Thanks for your participation. Let us begin!

Survey

First of all, we would like to know some of your socio-demographic characteristics, along with some information related to your health status and the blood donation process. And then we will ask you about a whole set of motivations and barriers for collaborative blood donation.

Table S.1.1. Socio-demographic and technological items

Variable	Values
Age	Continuous
Gender	Male / Female
Family situation	Live alone / live with partner / live with partner and children / other situations
Completed educational level	Primary / Secondary / University Graduate / Master or Doctorate
Work status	Entrepreneur or manager / qualified professional / manual worker / white-collar worker / student / retired or inactive / unemployed
Economic sector	Agriculture or livestock / industry / real estate / commerce / tourism / professional, financial and business services / health and well-being / education and research / public administration / others

Variable	Values
Type of locality	Village or rural area / small or medium city / large city or metropolitan area
Digital technology uses	On a scale of 1 to 5, where 1 is "never or almost never" and 5 is "very frequently, on a daily basis", indicate your degree of use of the following digital technologies, regardless of the device with which you do it (fixed computer or laptop, tablet, mobile phone ...):<br><br>- Browse and consult general content on the Internet / mobile apps<br>- Send and reply emails<br>- Participate in chats and social networks<br>- Participate in professional networks<br>- Obtain goods and services through collaborative platforms (accommodation, transport, services ...)<br>- Provide goods and services through collaborative platforms (accommodation, transport, services ...)<br><br>- Browse and consult health and wellbeing content on the Internet / mobile apps<br>- Send and reply emails related to health and wellbeing<br>- Participate in chats and social networks in the field of health<br>- Participate in professional networks in the field of health<br>- Obtain health-related goods and services through collaborative platforms<br>- Provide goods and services related to health through collaborative platforms

Table S.1.2 Health status and blood donation items

Variable	Question and values
Health status	How would you say your general health status is? (0 = not responding; 1 = very bad; 2 = bad; 3 = neither good nor bad; 4 = good; 5 = very good)
Disease/illness	Do you have a chronic illness or health problem? (0 = no answer; 1 = yes; 2 = no; 99 = don't know)
Closeness with the disease	Does anyone close to you have a chronic illness or serious health problem? (0 = no answer; 1 = yes; 2 = no; 99 = don't know)
Illness care	Are you in charge or care of a person who has a chronic illness or a serious health problem? (0 = no answer; 1 = yes; 2 = no)
Health system assessment	What would you say the health system –health centers, health professionals, and pharmacies- is in general in your locality? (0 = not responding; 1 = very bad; 2 = bad; 3 = neither good nor bad; 4 = good; 5 = very good).
Blood donation	Have you ever donated blood? (0 = no answer; 1 = yes; 2 = no)<br><br>If the person has answered YES (1):<br><br>- How many times would you say that you have donated in the last 5 years? (1 = once; 2 = between 2 and 5 times; 3 = between 6 and 10 times; 4 = more than 10 times);<br><br>- How would you rate your donation experience? (0 = not responding; 1 = very bad; 2 = bad; 3 = neither good nor bad; 4 = good; 5 = very good).<br><br>- What were the three main reasons that prompted you to donate blood? [Multiple response: maximum three options = response to a specific campaign / personal awareness / closeness to the disease / I have been a recipient of donated blood / helping others / convinced by a family member / convinced by friends / closeness to

Variable	Question and values
	the donation center / chance - I came across the donation center / other reasons (specify)]<br><br>- How do you think blood donation could be improved? [Multiple response: maximum three options = intensifying campaigns / using technologies better / raising awareness of donors / expanding the number of donation centers / extending donation hours / rewarding donors / improving the traceability of my donation (information about where my blood goes) / other reasons (specify)]<br><br>If the person has answered NO (2):<br><br>- What were the three main reasons that led you not to donate blood? [Multiple answer: maximum three options = I have not seen any specific campaign / lack of awareness towards donation / remoteness with the disease / I have not been a blood recipient / distrust of the donated blood management system / remoteness with the center of donation / not by chance - I have never come across the donation center /, I think the donation should be paid / fear of needles / fear of being ill after donation / other reasons (specify)]

Table S.1.3. Collaborative Blood Donation (CBD) Survey

Constructs	Items	Adapted from
Sharing economic benefits	CBD allows others to save money.<br>CBD allows others to lower my expenses.<br>CBD allows others to live thriftily.	[1], [2]
Uniqueness	My CBD gives others access unique product.<br>My CBD allows others to use unique product.<br>My CBD allows others to access products and services which cannot be found elsewhere.	[3]
Variety	CBD allows me to access a diverse range of donations.<br>CBD offers a large spectrum of donations and recipients.<br>CBD offers me a great diversity of donations and recipients.	[3]
Ubiquity and availability	CBD allows me to donate blood in many places.<br>CBD allows me to donate blood wherever I am.<br>CBD allows me to donate blood regardless of my location.	[3]
Social experience	I meet interesting people through CBD.<br>I get to know new people through CBD.<br>Through CBD I make nice acquaintances.	[3]
Risk-related concerns	Engaging in CBD constitutes an economic risk to me.<br>Engaging in CBD constitutes a legal risk to me.<br>You take a risk when engaging in CBD.	[3]
Privacy-related concerns	It is unpleasant that anyone can get insights into my private health sphere on CBD platforms.<br>It is unpleasant to disclose private data online for CBD.<br>It is unpleasant that many people can see my health private data on CBD platforms.	[4]
Scarcity-related concerns	CBD implies a high probability that a donation cannot be made when you want to make it.<br>CBD carries the risk that you cannot make the donation at any time.<br>In CBD, when you want to make a donation, it may not be available.<br>In CBD, donations are often not available when I want to make them.	[5]
Non-ownership prestige	People who give up their possessions have more prestige than those who do not give up.	[6]

Constructs	Items	Adapted from
	People who share their possessions have a high profile.<br>Giving or sharing possessions is a status symbol.	
Ownership-related independence	Ownership increases my independence from others.<br>Owning things myself makes me independent from other people.<br>Through ownership I gain independence from other people.	[3]
Ecological sustainability	CBD helps saving natural resources.<br>CBD is a sustainable mode of blood donation.<br>CBD is ecologically meaningful.<br>CBD is efficient in terms of using energy.<br>CBD is environmentally friendly.	[1]
Anti-capitalism	CBD allows me to not unnecessarily support large corporations.<br>CBD allows me to avoid capitalism.<br>CBD offers me an alternative to the capitalist system.	[5]
Community sense of belonging	I feel connected with others when using CBD.<br>I have a good bond with others in the CBD community.	[7]
Modern lifestyle	To me, CBD represents an up-to-date life style.<br>CBD meets the spirit of the age.<br>CBD is in tune with the times.	[3]
Effort expectations	It is cumbersome to participate in CBD activities.<br>I would have to familiarize with CBD a lot first.<br>It takes a long time to get acquainted to CBD.<br>CBD appears to be too circumstantial to me.	[8]
Trust in users and donors	Other CBD users are trustworthy.<br>Other CBD users keep promises and commitments.<br>Other CBD users usually keep my best interests in mind.	[9]
Attitude	Using CBD is a good idea.<br>Using CBD is a wise idea.<br>I like the idea of using CBD.<br>Using CBD is pleasant.	[10]
Subjective norm	People who are important to me think that I should participate in CBD.<br>People who influence my behavior think that I should participate in CBD.<br>People whose opinions I value prefer that I participate in CBD.	[8]
Perceived behavior control	I am able to use CBD.<br>Using CBD is entirely within my control.<br>I have the resources and the knowledge and the ability to make use of CBD.	[10]
CBD behavioral intention	I intend to use CBD in the future.<br>I will always try to use CBD in my daily life.<br>I plan to use CBD frequently.	[8]
CBD behavior	I am familiar with CBD.<br>I have experience with CBD.<br>I know a lot about how CBD actually works.	[5]

Items Measure (Scale 1 to 5; 1 = strongly disagree, 2 = disagree; 3 = neither agree nor disagree; 4 = agree; 5 = strongly agree)

References

1. Hamari J, Sjöklint M, Ukkonen A. The sharing economy: Why people participate in collaborative consumption. J Assoc Inf Sci Tech. 2016;67(9):2047-2059.

2. Lastovicka JL, Bettencourt LA, Hughner RS, Kuntze RK. Lifestyle of the tight and frugal: Theory and measurement. J Consum Res. 1999;26(1):85–98.
3. Hawlitschek F, Teubner T., Gimpel H. Consumer motives for peer-to-peer sharing. J Clean Prod. 2018;204:144-157.
4. Krasnova H, Günther O, Spiekermann S, Koroleva K. Privacy concerns and identity in online social networks. Identity Inf Soc. 2009; 2(1): 39-63.
5. Lamberton CP, Rose RL. When ours is better than mine? A framework for understanding and altering participation in commercial sharing systems. J Mark. 2012; 76(4):109–125.
6. Venkatesh V, Bala H. Technology acceptance model 3 and a research agenda on interventions. Decis Sci. 2008;39(2):273–315.
7. Peterson NA, Speer PW, McMillan DW. Validation of a brief sense of community scale: Confirmation of the principal theory of sense of community. J Community Psychol. 2008; 36(1):61-73.
8. Venkatesh V, Thong JYL, Xu X. Consumer acceptance and use of information technology : Extending the unified theory. MIS Q. 2012; 36(1):157-178.
9. Pavlou PA. Consumer acceptance of electronic commerce: Integrating trust and risk with the technology acceptance model. Int J Electron Commer. 2003; 7(3):69-103.
10. Taylor T, Todd PA. Understanding information technology usage: A test of competing models. Inf Syst Res. 1995; 6(2):144-176.
