# Supplementary material for: Using Digital Platforms to Promote Blood Donation: Motivational and Preliminary Evidence from Latin America and Spain
Source: Int J Environ Res Public Health. 2021 Apr 17;18(8):4270. doi: 10.3390/ijerph18084270 (PMC8073325; doi:10.3390/ijerph18084270)
Supplement: Supplementary file 1 [file ijerph-18-04270-s001.zip › Supplementary File S2.pdf]

Table S.2.1. Participants' characteristics, health, blood donation and sociodemographic issues

Health status	(302)	*Age*	(297)
Not responding	7.9	Under 30 years	8.2
Very bad	0.3	30 to 39 years	14.9
Bad	2.0	40 to 49 years	18.2
Neither good nor bad	14.6	50 to 59 years	24.3
Good	42.7	60 to 69 years	19.9
Very good	32.5	70 years and over	14.5
Health system assessment	(302)	*Gender*	(298)
Very bad	13.2	Male	54.7
Bad	19.2	Female	45.3
Neither good nor bad	33.8		
Good	26.8	*Human capital (education level)*	(284)
Very good	7.0	Primary	10.2
		Secondary	12.7
Blood donation	(302)	University graduate	40.1
Not responding	3.6	Master or doctorate	37.0
Yes	50.0		
No	46.4	*Occupational status*	(284)
		Self-employed and managers	20.4
5-years frequency of blood donation	(151)	Qualified professionals	47.5
Once	42.4	Manual workers	4.2
Between 2 and 5 times	47.7	Non-manual workers	9.2
Between 3 and 10 times	4.6	Students	7.4
More than 10 times	2.6	Retirees	7.2
		Unemployed	4.1
Blood donation experience	(151)		
Very bad	4.0	*Activity sector*	(284)
Bad	1.3	Agriculture/livestock	2.1
Neither good nor bad	13.2	Industry	8.8
Good	37.1	Real state	4.1
Very good	43.7	Commerce & Tourism	10.6
		Professional & business services	11.6
General digital daily uses	(284)	Health & well-being	18.0
Browse and consulting internet / apps	85.2	Education & research	27.5
Send and replay emails	72.2	Public administration	17.3
Participate in chats & social networks	70.4		
Participate in professional networks	38.0	*Household status*	(295)
P2P obtainer	25.4	Single-parent homes	21.3
P2P provider	18.3	Couple with children	28.8
		Couple without children	24.5
Health digital daily uses	(284)	Other situations	25.4
Browse and consulting internet / apps	30.6		
Send and replay emails	18.0	*Locality*	(296)
Participate in chats & social networks	13.7	Village or rural area	14.1
Participate in professional networks	13.0	Small or mid-sized town/city	54.9
P2P obtainer	9.9	Large town/city or metropolitan area	31.0
P2P provider	10.2		
		Country	(302)
		Chile	62.6
		Spain	11.2

Colombia	9.9
Puerto Rico	6.0
Other Latin America countries[1]	10.3

Notes: In brackets the number of participants. Valid percentages. 1. Distributed in small samples (from 2 to 5 participants) from Argentina, Bolivia, Costa Rica, Ecuador, Mexico, Peru, Uruguay and Venezuela.
