# Supplementary material for: Using Digital Platforms to Promote Blood Donation: Motivational and Preliminary Evidence from Latin America and Spain
Source: Int J Environ Res Public Health. 2021 Apr 17;18(8):4270. doi: 10.3390/ijerph18084270 (PMC8073325; doi:10.3390/ijerph18084270)
Supplement: Supplementary file 1 [file ijerph-18-04270-s001.zip › Supplementary File S3.pdf]

Table S.3.1. Model items, descriptive and correlation statistics

Construct/Items	Description	Mean	S.D.	Skewness	Kurtosis	VIF
Trust in others (TRU)						
TRU1	Other CBD users are trustworthy	3.00	0.055	-1.298	2.165	2.727
TRU3	Other CBD users usually keep my best interests in mind	2.98	0.054	-1.203	2.120	2.725
Modern lifestyle (MLS)						
MLS1	To me, CBD represents an up-to-date life style	3.75	0.068	-1.061	1.106	1.932
MLS3	CBD is in tune with the times	4.05	0.061	-1.583	1.988	1.937
Attitudes (ATT)						
ATT1	Using CBD is a good idea	4.12	0.061	-1.384	2.141	3.591
ATT2	Using CBD is a wise idea	3.94	0.063	-1.046	1.325	3.708
ATT4	Using CBD is pleasant	3.43	0.057	-0.408	1.230	1.554
Subjective Norm (SBN)						
SBN1	People who are important to me think that I should participate in CBD	3.15	0.064	-0.495	0.463	2.325
SBN3	People whose opinions I value prefer that I participate in CBD	3.09	0.059	-0.481	0.907	2.331
Perceived behavioral control (PBC)						
PBC1	I am able to use CBD	3.68	0.070	-0.969	0.702	1.731
PBC2	Using CBD is entirely within my control	3.20	0.073	-0.345	-0.467	1.732
Intention of collaborative donation (ICB)						
ICD1	I intend to use CBD in the future	3.66	0.069	-0.869	0.467	1.813
ICD2	I will always try to use CBD in my daily life	3.18	0.062	-0.482	0.391	1.810
Collaborative blood donation (CBD)						
CBD1	I am familiar with CBD	2.14	0.076	0.884	-0.384	2.684

CBD2	I have experience with CBD	1.81	0.069	1.334	0.944	2.687

Notes: CBS: Collaborative Blood Donation. N=302. Items measures based on 5-points Likert scale: 1 = strongly disagree; 2 = disagree; 3 = neither agree nor disagree; 4 = agree; 5 = strongly agree
